# Does comparison of self with others influence body image among adult women? An experimental study in naturalistic settings

**DOI:** 10.1007/s40519-021-01196-3

**Published:** 2021-04-26

**Authors:** Victoria Laker, Glenn Waller

**Affiliations:** Department of Psychology, University of Sheffield, Floor D, Cathedral Court, 1 Vicar Lane, Sheffield, S1 2LT UK

**Keywords:** Body image, Safety behaviors, Body comparison, Eating disorders

## Abstract

**Background and Objectives:**

It has been suggested that body comparison is a safety behavior in eating disorders. This experimental study investigates the causal impact of upward and downward body comparison on body image, eating pathology, self-esteem, anxiety and mood. It also considers whether trait body comparison and eating pathology are associated with responsiveness to upward and downward comparison.

**Methods:**

Thirty-nine women participated. Each completed trait comparison and eating pathology measures. Following this, each participant spent an hour (on different days) making an upward, downward or neutral comparison in a naturalistic setting. After each condition, the participant completed measures of body satisfaction, self-esteem, anxiety, depression and eating pathology.

**Results:**

Participants were significantly less satisfied with their bodies following upward comparison. Both upward and downward comparison were associated with particularly negative effects if an individual had greater trait eating concerns. The effects of downward comparison were correlated with increased anxiety.

**Limitations:**

The sample was lacking in diversity. Compliance with the experimental tasks was not strictly monitored.

**Conclusions:**

Upward comparison resulted in lower body satisfaction, but downward comparison did not result in positive effects. However, trait eating concerns and comparison influenced the impact of both forms of comparison. Body comparison should be a target for treatment in CBT for eating disorders, particularly where the individual has a strong tendency to make comparisons with other people.

**Level of evidence:**

Level III: Evidence obtained from well-designed cohort or case–control analytic studies.

## Introduction

Body comparison is the use of other people’s physical and related attributes to evaluate one’s own appearance relative to theirs [1]. Such comparison manifests in many patients with eating disorders [2]. Body comparison is hypothesised to be a safety behavior, which can have short-term positive outcomes but which maintains negative body image in the longer term. However, despite these negative implications, little is known about the mechanisms of body comparison. The few empirical studies that exist show that body comparison is associated with greater body dissatisfaction (e.g., [3–5]). However, that literature is largely correlational, meaning that the causality of any link cannot yet be established. Therefore, for example, it is possible that body dissatisfaction drives body comparison, rather than body comparison causing or maintaining body dissatisfaction. Some research has focused on social media and comparisons. One study found that people reported making more appearance comparisons following Facebook exposure than following looking at a control website [6]. However, most of the social media studies did not directly manipulate or measure comparison, therefore they are not relevant to this particular study, but it is worthy to note the importance that social media may play when making comparisons.

Social comparison theory [7] hypothesises that humans have an innate drive to evaluate their own opinions, abilities, progress, and standing in life. to fulfil this need, individuals identify standards against which they can compare themselves with others. Appearance is one of those factors, with social pressure for such comparison being particularly strong among women. Thus, women are particularly likely to engage in frequent comparisons with peers, judging their weight and shape in relation to others [8]. In Western society, the emphasis is on a slim female figure as being desirable, either due to social value placed on slimness [9], or possibly due to evolutionary pressure to attract mates [10]. Therefore, many women are likely to feel pressure to lose weight to achieve a more favourable comparison to their peers and other role models (e.g., people in the media) (e.g., [3, 11]).

Comparison can occur in two forms—upward or downward. With body image, upward comparison occurs when one compares oneself to someone who one believes is better than oneself (e.g., slimmer; with a more desirable body shape; with more pleasing hair). Downward body comparison occurs when one compares oneself to someone who one perceives to be worse than oneself (e.g., fatter; with a less desirable body shape; with poorer hair), though the possibility of doing this can be limited by having an inappropriately negative body image (as many women do). In the short term, downward comparison might relieve anxiety about appearance (e.g., weight), resulting in a greater sense of status. In contrast, upward comparison is likely to lead to a long-term increase in anxiety and lower perceived status.

In summary, social comparison theory makes different predictions about the outcomes of upward and downward body comparisons. However, the literature to support these hypotheses is limited. An extensive literature review [12] found that body comparison leads to higher body dissatisfaction, but did not consider the difference between upward and downward comparison. This field has been limited in quantity and by its almost exclusively correlational nature, but indicates that upward comparison is associated with a wide range of psychological disturbances, including eating pathology [13, 14], body dissatisfaction [14–18], and negative affect [14, 18]. However, no studies have looked at downward comparison on its own, and very few contrast the effects of upward and downward comparison directly. Studies that do so have had mixed results. In two experimental studies, upward comparison resulted in more body dissatisfaction, negative affect and guilt (as above), whereas downward comparison had the opposite effect on all three [8, 19]. In contrast, a correlational study [20] found that women who engaged in comparison were more likely to display drive for thinness behaviors, more body dissatisfaction and more dietary restraint. While, this result was found for both upward and downward comparison, the correlation was stronger with upward comparison.

Given the potential negative impact of comparison, and also the lack of data differentiating upward and downward comparison, a greater understanding of the causal role of upward and downward comparison is needed. This understanding will help to plan and deliver interventions for those who experience body image issues and related disorders. However such research would be more conclusive if it were experimental, as so far the research has mainly been correlational. Therefore, the aim of this study is to establish whether upward and downward body comparisons drive how an individual feels about themselves (body image, eating pathology, and other concerns), and to determine whether there are traits that influence any such effects. It is hypothesised that upward comparison will have negative consequences relative to neutral comparison (where neutral comparison is defined as ‘normal’ comparison—i.e., the participant is not given any instructions, so will make comparisons as is normal for them), while downward comparison will result in positive outcomes for the individual. Supplementary analyses will consider whether trait comparison and eating pathology are associated with more negative responses to body comparison.

## Methods

### Ethics

This study was reviewed and approved by the Ethics committee of the Psychology Department of the University of Sheffield.

### Design

This study used a within-subjects naturalistic experimental design. The within-subjects design was selected because fewer participants are required to gain a large effect size and because recruitment would be easier if fewer participants were needed, due to the long length of the study. This method also allows for direct comparisons between and within the participants for the different types of comparison. The dependent variables are the measures of pathology (anxiety, depression, self-esteem, eating pathology and body satisfaction). The independent variable is the three conditions–upward comparison, downward comparison, and neutral (i.e., no focus on comparison).

### Participants

A sample of 39 female adults completed the study (mean age = 20.47 years; *SD* = 1.09; range = 18–55 years). Most were Caucasian (*N* = 30; South Asian = 2; East Asian = 4; mixed ethnicity = 3). Sample size analysis was carried out using G-Power, which demonstrated that 36 participants were required for a power of 80%, a medium effect size, and *P* < 0.05. Thus, the study was adequately powered. The participants were recruited from a Psychology course, and received credits for participation.

### Measures

The following measures were used to assess comparison, eating behaviors and cognitions, levels of anxiety and depression, and body satisfaction. The instructions for the measures were amended (where required) to ask how the participants were feeling right now (i.e., after completing the required comparisons).

#### 2.4.1 Comparison of self survey (CoSS, [1])

The CoSS is a 22-item self-report measure, with two factors—‘Physical Appearance’ and ‘Personality’. The ‘Physical Appearance’ factor measures how much the individual compares their body and other physical aspects with others. The ‘Personality’ factor measures how much the individual compares their personality with others. The higher the score on CoSS, the more comparison an individual makes. The CoSS has good psychometric properties. It has excellent test–retest reliability (*r* = 0.93, *r* = 0.90; for the ‘Physical Appearance’ and ‘Personality’ comparison factors respectively). It also has strong internal consistency (Cronbach’s *alpha* = 0.916 and 0.891 for the ‘Physical Appearance’ and ‘Personality’ comparison factors, respectively). Finally, it has good concurrent validity, correlating strongly (*P* < 0.001) with another measure of comparison–the Body, Eating, and Exercise Comparison Orientation Measure [21].

#### 2.4.2 Eating disorder examination questionnaire (EDE-Q, [22])

The EDE-Q is a 28-item self-report measure of eating disordered cognitions and behaviors. Higher scores indicate a greater level of eating pathology. The scores for each factor were calculated, as well as the Global eating pathology score. The EDE-Q has limited psychometric properties, but has good clinical utility with clinical and non-clinical populations [23].

#### 2.4.3 ED-15 [24]

The ED-15 is a 15-item self-report measure of eating-disordered cognitions and behaviors. For the purpose of this study, only the 10 attitudinal questions were used. These questions form two factors–Weight and Shape, and Eating. Higher scores indicate a greater level of eating pathology. The scores for the two factors were calculated, as well as an overall eating pathology score. The ED-15 has strong psychometric properties, including test–retest reliability (*r* = 0.85–0.93), concurrent validity (*r* = 0.56–0.89), and convergent validity (*r* = 0.31–0.63). The ED-15 also shows good clinical validity [24].

#### 2.4.4 Generalized anxiety disorder questionnaire (GAD-7, [25])

The GAD-7 is a seven-item self-report measure, used for screening and measuring the severity of anxiety. Higher scores indicate a higher level of anxiety. An overall anxiety score was calculated. The GAD-7 has satisfactory psychometric properties including internal consistency (α = 0.92), test–retest reliability (*r* = 0.83), and concurrent validity (*r* = 0.72–0.74; [25]). Clinical validity is good in a general and primary care population [26]. Convergent validity is strong (*r* = 0.74–0.75; [27]).

#### 2.4.5 Patient health questionnaire (PHQ-9, [28])

The PHQ-9 is a nine-item self-report measure of depression, which is used widely within clinical settings for screening and the measurement of outcome. Higher scores indicate a higher level of depression. An overall depression score was calculated. The PHQ-9 has well-established psychometric properties, including good internal validity (Cronbach’s alpha = 0.86–0.89). Clinical validity within the general and primary care population is excellent, as is its convergent validity [28].

#### 2.4.6 Body Satisfaction Scale (BSS, [29])

The BSS is a 16-item self-report measure, which determines the individual’s level of satisfaction with their body. There are two factors – head and body. Higher scores indicate a lower level of body satisfaction. Scores for the two factors were calculated, as well as an overall satisfaction score. The BSS has satisfactory internal consistency (α = 0.79 – 0.89).

#### 2.4.7 Rosenberg self-esteem scale (RSE, [30])

The RSE is a 10-item self-report measure of self-esteem. Higher scores indicate a lower level of self-esteem. An overall self-esteem score was calculated. The RSE has excellent test re-test reliability (*r* = 0.85–0.88), and internal consistency (α = 0.77–0.88).

### Procedure

The participants were recruited online using the University Psychology recruitment system. Undergraduate Psychology students sign up for studies advertised on the website in return for credits required to complete the course. The study was delivered via an online system. An explanation of the study was provided before the participant consented. The information specified female participants only. Once the student consented, they were provided with a link to the initial questionnaires. All measures were accessed via Qualtrics survey software. At this stage, if any males had signed up (*N* = 14) they were filtered and removed from the final sample.

Once the participant completed the initial questionnaires, they were sent the first set of instructions. Each participant completed all three conditions (order counter-balanced) on three consecutive days. In the upward comparison condition, the participants were asked to make a comparison with someone they could see (or had seen recently) who they thought had a better body than them, and think about all the ways in which that person looks better than them. The participant was advised the comparison should only take a couple of seconds but to make a comparison every five minutes for one hour. Following this hour, the participant was asked to follow a link in the instructions to complete the five measures. In the downward comparison condition, the participants were asked to make a comparison with someone who they could see (or had seen recently) who they thought had a worse body than them, and to think about all the ways in which that person looks worse than them. Again, the participant was asked to take only a couple of seconds but to make a comparison every five minutes for one hour. Again, the participant then followed a link in the instructions to complete the five measures. For both of these active conditions, the participant was advised to set an alarm to go off every five minutes if they thought they may forget to complete the task, and was asked to do the comparison as close to the five minutes as possible. In the neutral condition, the participant was asked to continue with their day as they normally would, and at a time suitable to them to follow a link to complete the measures. Once all three conditions had been completed, the participants were sent a debrief informing them of the nature of the study. The completion times were checked, to ensure the participant had spent the relevant time period on each phase. Ninety individuals activated the link, 71 completed the primary measures, and 39 completed the whole experiment (completion rate = 43.33%).

### Data analysis

A repeated measures ANOVA was used to determine the impact of the three conditions (upward comparison, downward comparison, neutral) on body image, eating concerns, anxiety, depression and self-esteem. Effect sizes were reported as partial *eta*^*2*^, where a score of > 0.14 indicates a large effect. Pearson’s correlation coefficients were used to test associations between trait characteristics (comparison; eating pathology) and responsiveness to upward and downward comparison (relative to the neutral condition).

## Results

### Characteristic comparison of completers versus non-completers.

The overall completion rate was low (43%). The age range most likely to complete the whole study were the 60 + group (100%), although this was also the group with the smallest number of participants. Caucasian participants accounted for 75.00% of the sample but had a low completion rate (66.67%), while mixed-race participants were the highest completers (100.00%). The least likely to complete were South Asian participants (50.00%). Those participants previously diagnosed with an eating disorder were less likely to complete than those who have not been previously diagnosed (20.00% versus 71.70%). Participants were more likely to complete the study if they were older, and had not previously had an eating disorder. Therefore the results are likely to be skewed towards this group and not necessarily generalizable to others.

### Impact of different types of body comparison

Table 1 shows levels of body image, eating pathology, anxiety, depression and self-esteem following upward comparison, downward comparison, and the neutral condition (i.e., the participant’s normal level of comparison).

**Table 1:** Mean scores and ANOVA results on measures of body satisfaction, eating pathology, self-esteem, depression and anxiety

		Condition			ANOVA			
		Neutral	Upward	Downward	*F*	*P*	LSD	Partial eta^2^
*ED-15 scales*								
Weight & Shape	*M*	2.75	3.05	2.90	1.54	*NS*	–	–
	*(SD)*	(1.19)	(1.50)	(1.39)				
Cognition	*M*	2.63	2.97	2.81	1.86	*NS*	–	–
	*(SD)*	(1.19)	(1.39)	(1.30)				
*GAD-7 scales*								
Anxiety	*M*	1.87	1.95	1.88	0.91	*NS*	–	–
	*(SD)*	(0.51)	(0.55)	(0.65)				
*PHQ-9 scales*								
Depression	*M*	1.90	1.95	1.93	0.34	*NS*	–	–
	*(SD)*	(0.51)	(0.51)	(0.59)				
*BSS scales*								
Head	*M*	3.06	3.14	2.97	1.17	*NS*	–	–
	*(SD)*	(1.18)	(1.15)	(1.19)				
Body	*M*	3.58	3.88	3.65	5.71	0.01	D = N < U	0.24
	*(SD)*	(1.31)	(1.21)	(1.38)				
*RSE scales*								
Self-esteem	*M*	2.35	2.36	2.33	0.21	*NS*	–	–
	*(SD)*	(0.30)	(0.29)	(0.38)				

Repeated measures ANOVAs showed that there were no effects of comparison type on eating attitudes, anxiety, depression, head satisfaction or self-esteem. However, there was a significant effect of body comparison on body satisfaction. Participants were significantly less satisfied with their bodies following upward comparison than after neutral or downward comparison. This difference showed a very large effect size (partial *eta*^*2*^).

### Association of the effects of body comparison with trait characteristics

To determine whether trait comparison and eating pathology are associated with responsiveness to upward or downward comparison, change scores were used to represent the difference in scores between the two conditions. Change scores were calculated by subtracting the score on the neutral condition from the score on the active condition. Thus, a positive 'upward' change score would indicate a greater level of the key variable (e.g., body dissatisfaction) after undertaking upward comparison, while a negative 'downward' change score would indicate less of that variable after undertaking downward comparison.

To give context of how representative the sample is of people with eating pathology, the mean and standard deviation for the EDE-Q was calculated (*M* = 2.74, *SD* = 1.26). This mean is high relative to the mean for purely non-clinical groups, and is near to the clinical cut-off suggested by Fairburn for UK women.

Table 2 shows whether trait comparison and eating pathology are associated with responsiveness to upward comparison. *P* values of 0.01 and 0.001 were adopted, to reduce the risk of type I errors. EDE-Q Eating Concerns was the trait measure that displayed the most consistent pattern of associations with response to upward comparison. Greater levels of trait eating concerns were strongly associated with negative reactions to upward comparison in terms of eating pathology, head satisfaction and body satisfaction. Both scales of the CoSS and trait EDE-Q Weight Concerns were associated with changes in response to upward comparison. Greater levels of trait comparison and weight concerns were strongly associated with negative reactions to upward comparison in terms of ED-15 weight concerns.

**Table 2:** Pearson’s correlation (*r*) between the change in score on CoSS and EDE-Q scales following upward comparisons and neutral conditions

	CoSS Scale		EDE-Q			
	Physical appearance	Personality	Restraint	Shape concerns	Eating concerns	Weight concerns
GAD anxiety	0.15	0.16	0.18	0.21	0.34	0.21
PHQ depression	0.10	0.19	– 0.04	0.09	0.24	0.11
ED-15 weight	0.47*	0.45*	0.35	0.38	0.61**	0.42*
ED-15 cognition	0.39	0.38	0.31	0.23	0.55**	0.27
BSS head	0.16	0.20	0.06	– 0.03	0.53**	0.10
BSS body	0.21	0.26	0.22	0.14	0.62**	0.25
RSE self esteem	0.18	0.22	0.21	0.17	0.34	0.21

*P < 0.01, **P < 0.001

Table 3 shows whether trait comparison and eating pathology are associated with responsiveness to downward comparison. As with upward comparison, the largest number of correlations were with trait EDE-Q eating concerns. Individuals with greater EDE-Q Eating Concerns were influenced more strongly by downward comparison, which was associated with greater ED-15 scores, body dissatisfaction and anxiety following downward comparison. The CoSS scales and trait EDE-Q Restraint behavior were also associated with increases in ED-15 weight concerns following downward comparison. Higher levels of EDE-Q Restraint also correlated positively with higher levels of BSS body dissatisfaction following downward comparison.

**Table 3:** Pearson’s correlation (*r*) between the change in score on CoSS and EDE-Q scales following downward comparisons and neutral comparison conditions

	CoSS Scale		EDE-Q			
	Physical appearance	Personality	Restraint	Shape concerns	Eating concerns	Weight concerns
GAD Anxiety	0.23	0.27	0.24	0.07	0.47*	0.10
PHQ depression	0.23	0.30	0.05	0.08	0.24	0.05
ED-15 weight	0.46*	0.50**	0.41*	0.25	0.67**	0.31
ED-15 cognition	0.31	0.38	0.24	0.17	0.54**	0.18
BSS head	0.38	0.37	0.23	0.19	0.31	0.13
BSS body	0.31	0.31	0.44*	0.26	0.61**	0.24
RSE self esteem	0.18	0.22	0.21	0.17	0.34	0.21

*P < 0.01, **P < 0.001

## Summary

Overall, this experimental study has shown that upward comparison has negative effects, specifically on individuals’ body satisfaction. However, comparison has particularly negative effects if an individual has trait eating concerns. Downward comparison has negative effects if one has trait eating concerns, restraint and comparison behaviors. Downward comparison is correlated with higher anxiety levels and weight and shape concerns. This finding is similar to upward comparison, with the exception that upward comparison did not correlate with an increase in anxiety.

## Discussion

The aim of this study was to establish whether upward and downward body comparisons have different influences on the way an individual feels about themselves (body image, eating pathology, and other concerns). There was such a difference overall, but it was specific to body image. Upward comparison resulted in less satisfaction with one’s body. Considering the supplementary analyses, trait eating concerns and comparison behavior were a particular risk for people who make comparisons. Participants with stronger eating concerns are more likely to experience negative reactions to upward and downward comparison in terms of eating attitudes and body dissatisfaction.

The current results have similarities with existing correlational and experimental studies [8, 19, 20]. In the current study, upward body comparison had negative effects on the individual. Downward body comparison was also found to have negative effects, but only if the individual had trait eating concerns. The negative outcome of upward comparison is consistent with the existing empirical evidence [8, 19, 20], which shows similar negative effects. This consistent evidence supports the conclusion that upward body comparison is a safety behavior with negative short and long-term consequences. Given that such comparison is a very common phenomenon (e.g., in the use of social media), it clearly could play a strong potential role in maintaining or worsening normative body dissatisfaction. In contrast, the small existing literature is unclear about downward comparison. Some studies show that downward comparison has positive effects on body image [8, 19], whilst one shows downward comparison as being related to poorer body image [20]. The current study gives a potential reason for this inconsistency in findings – the characteristics of the participants in the studies. It is possible that those studies differed in their participants’ trait eating concerns, potentially explaining their contrasting effects. Future research should consider whether individual characteristics influence results in this way. That research might also consider why it was trait eating concerns rather than trait shape and weight concerns that had this effect.

This study has a number of limitations. While its experimental design has allowed robust conclusions to be reached, the study those have to be considered in light of those limitations. The sample consisted of young, adult females with limited ethnic diversity, limiting the generalizability of the results. The possibility of age, gender and ethnicity influencing these findings should be explored in further studies. Such research should also monitor compliance with the experimental tasks, to ensure that the participants focus on the core task. The completion rate for the study was fairly low (43%), probably due to the fairly demanding nature of the study (one hour each on three consecutive days). However, it is possible that drop out will have skewed the findings, as those who completed the study might have specific characteristics. For example, participants from undergraduate Psychology courses were given credits that are required by the University, making them more likely to complete the research Similarly, it is possible that the low completion rate resulted in Type 2 errors, due to limitations on power to find small changes and associations. Furthermore, it cannot be assumed that these findings will necessarily relate to a group with eating disorders, as the patterns of association might be specific to this type of sample. The long-term nature of the study might have encouraged those with a specific interest in body image to persevere, whilst others dropped out, again limiting generalisability.

Future research should consider the domain of comparison made. The participants were asked to make comparisons based purely on physical attributes. However, personality comparison also plays a significant role [1]. Therefore, the effects of both personality and physical comparison tasks should be considered. Those studies should also consider whether the object of comparison (e.g., relative to a person or to another representation) is critical.

This study has a number of potential clinical implications. it might be beneficial for clinicians to routinely assess whether the patient makes body and other types of comparisons, either at interview or using a standardised measure of comparison, such as the CoSS [1]. Any such tendency to use comparisons could then be considered within case formulations, to explain their positive and negative maintaining effects in terms of beliefs, emotions and behaviors. If the results of this study are replicated in clinical samples, then beliefs about the value of such comparison (and alternatives) can be generated and tested through experimenting with comparing vs not comparing [31]. Thus, the individual can learn that the safety behavior of comparison is associated with long-term negative outcomes that outweigh any short-term benefits.

### Strengths and limits

The strengths of this study lie in its experimental nature, in a field where mainly correlational work has been completed. This design allows for clearer causal conclusions. This study also considers the influence of trait comparison and eating behaviours on the outcome of comparison. The study does have some limitations, such as a homogenous sample. The naturalistic nature of the study also means that there is likely to be a level of variance due to differences in compliance. There was also a high drop-out rate due to the demands on participants’ time, so generalizability could be an issue.

## What is already known on this subject?

It is known that comparison is a normal human behaviour, but becomes a problem when it is conducted out of context or done excessively. Previous research has shown that excessive body comparison is associated with greater body dissatisfaction. Social comparison theory suggests that there are two types of body comparison—upwards and downwards—which should result in different outcomes.

## What does this study add?

The negative impact of upward body comparison is supported. However, this study shows that downward comparison has a negative impact if an individual displays trait eating concerns. The findings confirm that clinicians should routinely assess whether patients make body comparisons, and build them into case formulations.

## Data Availability

The data will be made available upon request.
